# The Impact of Heavy Smoking on Male Infertility and Its Correlation with the Expression Levels of the *PTPRN2* and *PGAM5* Genes

**DOI:** 10.3390/genes14081617

**Published:** 2023-08-12

**Authors:** Houda Amor, Yaser Alkhaled, Riffat Bibi, Mohamad Eid Hammadeh, Peter Michael Jankowski

**Affiliations:** 1 Department of Obstetrics, Gynecology and Reproductive Medicine, Saarland University Clinic, 66424 Homburg, Germany; 2 Department of Animal Sciences, Faculty of Biological Sciences, Quaid-i-Azam University Islamabad, Islamabad 44000, Pakistan; riffat.skmc@gmail.com

**Keywords:** male infertility, heavy smoking, transcript level, *PGAM5*, *PTPRN2*, *TYRO3*

## Abstract

Smoking has been linked to male infertility by affecting the sperm epigenome and genome. In this study, we aimed to determine possible changes in the transcript levels of *PGAM5* (the phosphoglycerate mutase family member 5), *PTPRN2* (protein tyrosine phosphatase, N2-type receptor), and *TYRO3* (tyrosine protein kinase receptor) in heavy smokers compared to non-smokers, and to investigate their association with the fundamental sperm parameters. In total, 118 sperm samples (63 heavy-smokers (G1) and 55 non-smokers (G2)) were included in this study. A semen analysis was performed according to the WHO guidelines. After a total RNA extraction, RT-PCR was used to quantify the transcript levels of the studied genes. In G1, a significant decrease in the standard semen parameters in comparison to the non-smokers was shown (*p* < 0.05). Moreover, *PGAM5* and *PTPRN2* were differentially expressed (*p* ≤ 0.03 and *p* ≤ 0.01, respectively) and downregulated in the spermatozoa of G1 compared to G2. In contrast, no difference was observed for *TYRO3* (*p* ≤ 0.3). In G1, the mRNA expression level of the studied genes was correlated negatively with motility, sperm count, normal form, vitality, and sperm membrane integrity (*p* < 0.05). Therefore, smoking may affect gene expression and male fertility by altering the DNA methylation patterns in the genes associated with fertility and sperm quality, including *PGAM5*, *PTPRN2*, and *TYRO3*.

## 1. Introduction

Nearly 15% of infertile couples worldwide are attributable to the male factor [1]. Several factors contribute to male infertility. Reproductive tract infections, genetic and anatomical disorders, and immunological and endocrine disorders are among these factors [2,3,4].

Lifestyle and environmental factors such as diet, smoking, exercise, and alcohol consumption are as important to our health as our genes. These factors cause changes that affect gene expression.

Gene expression is regulated in several ways in mammals. However, DNA methylation is the most common epigenetic signaling device that cells use to lock or unlock genes. DNA methylation works by adding a methyl group at position 5 of cytosine, which is found in cytosine phosphate guanine dinucleotides “CpGs” [5]. Typically, this group is added to specific sites in the DNA, where it blocks proteins that bind to the DNA to “read” the gene. This group can be removed through a process called demethylation. Typically, methylation turns genes “off” and demethylation turns genes “on” [5]. In recent decades, DNA methylation has received considerable research attention due to its importance in various cellular processes. DNA methylation plays a crucial role in regulating gene expression by regulating the transcription of DNA to RNA [6,7], and is involved in protecting the genome by inhibiting the movement of DNA transposable elements’ integrity [8,9]. DNA methylation processes can also be altered by many other factors, such as genetics [10] and environmental factors, smoking being a typical example [11].

About 46% of men of reproductive age, aged 20 to 39, are smokers [12]. Almost 75% of men who smoke daily live in countries with a medium or high Human Development Index [13]. In the USA, smoking is more common among men, with an overall prevalence of 20.5% and 21.9% in the 25 to 44 age group [14].

There are conflicting results regarding the effects of tobacco smoke on the standard semen parameters. Some studies have found that smoking is associated with decreases in sperm volume, sperm count, motility, and semen morphology [15,16,17,18]. Sharma et al. (2016) [19] demonstrated that smoking often negatively affects the standard semen parameters, and this is more pronounced in infertile male patients than normal populations, since their sperm may be more sensitive to inhaled toxic chemicals [20]. However, others have not found a meaningful effect of smoking on the conventional sperm parameters [21,22,23].

Cigarette smoking increases harmful oxidants such as reactive oxygen species (ROS) in the seminal plasma. This leads to an imbalance between antioxidants and oxidants, which is known as oxidative stress. Oxidative stress can lead to damage to sperm DNA [24,25,26]. In addition, studies have shown that sperm DNA damage may also be caused by the formation of DNA adducts associated with cigarette content [27,28]. This may affect the sperm epigenome and genome [29], and possibly the developing embryo [30,31].

In a previous study, we focused on three genes: *PGAM5* (phosphoglycerate mutase family member 5), *PTPRN2* (protein tyrosine phosphatase, N2-type receptor), and *TYRO3* (tyrosine protein kinase receptor). The *PGMA5* gene encodes two mitochondrial genes: *PGAM5s* and *PGMA5L* (phosphoglycerate mutase family member 5) [32]. Both proteins are expressed in the testes and play an important role in mitophagy, a cellular process that eliminates damaged mitochondria [33,34]. *PGAM5* might modulate the activity of PINK1 (PTEN-induced putative kinase 1) and Parkin, key proteins involved in mitochondrial quality control and autophagy [33,34]. Thus, a disruption of PGAM5 could lead to abnormal mitochondrial function in sperm, impacting their ability to fertilize oocytes. The second gene is *PTPRN2*, encoding a protein called tyrosine phosphatase non-receptor type 2, which is involved in the regulation of insulin signaling and glucose metabolism [32]. This pathway has been linked to male fertility through its impact on glucose metabolism in the testes. A previous study found that the semen parameters (semen volume, sperm concentration, motility, and morphology) were reduced in diabetic patients compared to non-diabetic patients [35]. The third gene is *TYRO3*, encoding a receptor tyrosine kinase protein [32]. This protein is expressed in the male reproductive system and might be involved in the regulation of Sertoli cells, which support the development of sperm cells in the seminiferous tubules [36,37,38].

The results of this study showed that cigarette smoking has an influence on DNA methylation levels. After applying bisulfite sequencing, a significant increase in the DNA methylation levels was observed between smokers and non-smokers: at 15 CpG sites in the *PGAM5*-gene-related amplicon and 9 CpG sites in the *PTPRN2*-gene-related amplicon. On the contrary, the results showed that the DNA methylation levels at three CpG sites in the *TYRO3*-gene-associated amplicon were significantly reduced in the case group compared to the control group [39]. Furthermore, this study showed a significant correlation between changes in sperm DNA methylation levels and standard sperm parameters in the case cohort [39].

Therefore, in this study, we aimed to determine possible changes in the transcript levels of the *PTPRN2*, *PGAM5*, and *TYRO3* genes in heavy smokers compared to non-smokers, and to investigate their association with the fundamental sperm parameters.

## 2. Materials and Methods

### 2.1. Semen Sample Collection

Before the beginning of this study, an institutional review board approval (No. PHRC/HC/13/14) was obtained from the Ethics Committee of Helsinki. Moreover, approval consent was taken from each participant enrolled in this study. The study was conducted in the laboratory of biochemistry and molecular biology of reproductive medicine, the Department of Obstetrics and Gynecology at the University Hospital-Homburg, Saarland, Germany.

All participating males were in the reproductive age group (25–45 years old) and the following parameters were excluded: cases suffering from varicocele, anti-sperm antibodies, Y chromosome microdeletions, males subjected to a surgical operation on the reproductive system, those with a heavy body mass index, and patients with any metabolic disorders.

The patients were classified into two groups. The control group included 55 proven fertile candidates who showed no previous history of smoking, and the case group included 63 fertile heavy smokers who smoked more than 20 cigarettes per day (in the last 3 years until their enrolment in this study). Semen samples were collected via masturbation after 3 to 5 days of sexual abstinence in clean, dry, sterile, and leak-proof plastic containers in a collection room attached to the laboratory. Following the liquefaction of the semen samples at 37 °C for 30 min to 1 h, the samples were analyzed according to the World Health Organization guidelines [40].

### 2.2. Spermatozoa Purification and Analysis

The samples were kept in the heating stage for 30 min for liquefaction at 37 °C. After that, the sperm samples were evaluated according to the WHO laboratory manual to determine the sperm count, motility, morphology, vitality (Eosin test), and sperm membrane integrity (Hos test) [40].

Gradient centrifugation was used to purify the samples. Briefly, each sample was treated using a discontinuous Puresperm gradient (40–80%) (Nidacon International, Mölndal, Sweden). Then, to guarantee the elimination of somatic cells, the samples were washed with lysis buffer (0.1% SDS, 0.5% Triton X-100 in double-distilled water).

#### 2.2.1. Assessment of Sperm Morphology

The sperm morphology was evaluated according to strict criteria, as follows:

Smears were prepared by spreading 20 μL of ejaculate on a glass slide. After fixation, the slides were stained using the Papanicolaou method. A total of 200 spermatozoa from each slide were evaluated under oil immersion at a magnification of 1000× using bright-field illumination. At least 10 high-power fields from different areas of the slide were estimated.

#### 2.2.2. Assessment of Sperm Vitality (Eosin Test)

**Figure 1:**
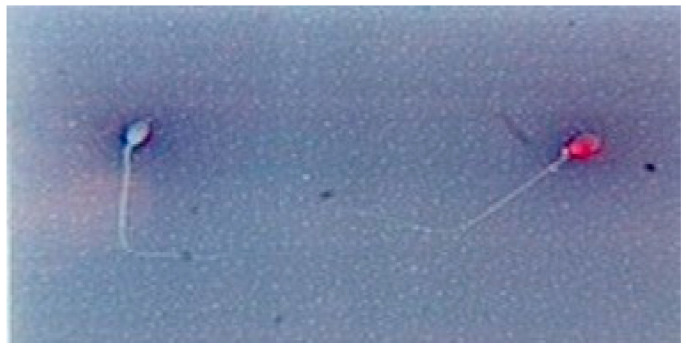
Vitality assessment of spermatozoa (Eosin test): unstained (white) alive spermatozoa and stained (red) dead spermatozoa.

On a glass slide, 10 μL of ejaculate was mixed with 10 μL of 0.5% aqueous yellowish Eosin Y solution. The mixture was covered with a cover slide, then evaluated after 3–5 min by distinguishing between the dead spermatozoa (red stained) and live spermatozoa (not stained) (Figure 1). A total of 200 spermatozoa from each slide were evaluated per slide under a phase-contrast microscope.

#### 2.2.3. Assessment of the Sperm Membrane Integrity (Hypo-Osmotic Test (HOS))

**Figure 2:**
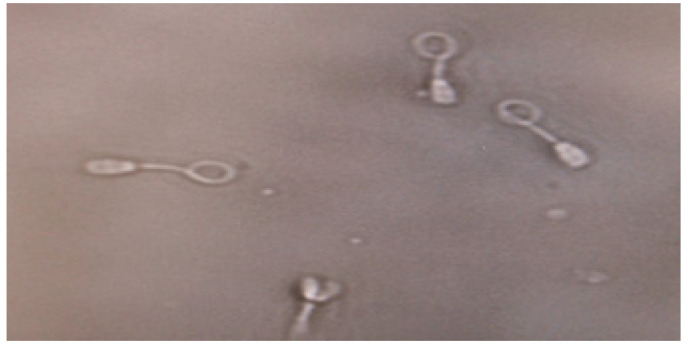
Hypo-osmotic swelling test (HOS test): tail swelling (normal spermatozoa) and no tail swelling (abnormal spermatozoa).

The HOS test is a sperm vitality test that predicts sperm membrane integrity. For this test, 100 μL of fresh ejaculate was mixed with 1.0 mL of a hypo-osmotic solution. Then, the mixture was incubated at 37 °C for 30–60 min. The influx of the fluid due to hypo-osmotic stress caused the sperm tail to swell, which indicated the presence of sperm with a functional and intact plasma membrane. A minimum of 200 swollen and/or not swollen spermatozoa were examined per slide under a phase-contrast microscope (Figure 2).

### 2.3. RNA Extraction and Synthesis of the cDNA (Reverse Transcription)

The total RNA was isolated from the purified semen samples according to a modified protocol of the Isolate II RNA/DNA/Protein Kit (Phenol-free) (Bioline, London, UK). The Nanodrop spectrophotometer ND-2000c (Thermo Scientific, Waltham, MA, USA) was used to measure the concentration and purity of the isolated RNA. The integrity of the isolated RNA was checked on an RNA Nano 6000 chip via an Agilent Bioanalyzer (Agilent technologies, Santa Clara, CA, USA). The extracted RNA was stored at −80 °C until the time of usage.

The reverse transcription procedure was performed using a miScript II RT Kit according to the standard protocol provided by the manufacturer (Cat No. 218161, Qiagen, Hilden, Germany). Briefly, 300 ng of extracted RNA was added to 4 μL of 5× MiScriptHiLflex buffer, 2 μL of nucleotide mix, and 2 μL of reverse transcriptase mix, which contained all the components required for the synthesis of the complementary DNA (cDNA). Then, RNA-free water was added to complete the required volume. After that, the reaction buffer was incubated at 37 °C for 60 min, then at 95 °C for 5 min to suppress the activity of the reverse transcriptase mix.

### 2.4. Real-Time Quantitative PCR (qPCR)

After the synthesis of the cDNA, real-time qPCR was performed to amplify and quantify the transcript level of each of the studied genes: *PGAM5*, *TYRO3*, *PTPRN2*, and GAPDH (as an endogenous control).

Briefly, the produced cDNA was used as the template for the qPCR reaction mixture. This reaction was prepared using 2× quantiTect SYBR Green PCR Mix (Cat No. 204143, Qiagen, Germany) and a QuantiTect primers assay for *PGAM5*, *TYRO3*, *PTPRN2*, and GAPDH (as a reference gene) (Cat No. QT00079247, Qiagen, Germany), according to the recommendations of the manufacturer.

At the end, the prepared mixture was distributed in triplicate for each sample into 96-well plates. The plates were placed in a StepOnePlus™ System (7500 Fast Applied Biosystems, Waltham, MA, USA) and the appropriate program was applied, according to the instructions provided by the manufacturer.

In addition, a no template control (NTC) and a no reverse transcriptase control (NRT) were included in each run. All the qPCR experiments were performed in triplicate and the resulting CT values were normalized to GAPDH.

### 2.5. Statistical Analysis

The quantification of the gene expression was determined through the CT value (threshold cycle), which was provided by a Real-Time PCR instrument with the software when the PCR reaction reached the start of its exponential stage and was imported into a spreadsheet program such as Microsoft Excel. The average of the Ct for each triple sample was calculated, the change in the target gene expression and the Cт average of each triple sample were determined, then the expression of the target and control genes were normalized to the endogenous gene by ΔCт (Cт target gene − Cт GAPDH) and (Cт control − Cт GAPDH). The ΔΔCт was calculated using (ΔCт target − ΔCт control) and the fold change in gene expression was calculated using the equation fold change = 2 − ΔΔCт [41].

For the data analysis, IBM SPSS for Windows software package version 23.0 (SPSS Inc., Chicago, IL, USA) was used. The samples included in this study were non-normally distributed (non-parametric) according to the values of the skewness test, Kurtosis test, Z-value, and Shapiro test. The independent sample *t*-test (Mann–Whitney test) was used to compare the means of the quantitative variables.

In addition, Spearman’s test was used to assess the correlation coefficient between the expression levels in the fertile heavy smokers and sperm parameters. To be qualified as statistically significant, the results should show a *p*-value less than 5% (*p* ≤ 0.05).

## 3. Results

One hundred and eighteen sperm samples were divided into two groups. The first group included 55 proven fertile candidates who showed no previous history of smoking as a control group, with a mean age of 36.33 ± 6.18. The second group included 63 fertile smokers who smoked more than 20 cigarettes per day, with a mean age of 37.42 ± 5.24, as the case group.

### 3.1. Characteristics of the Study Population

The sperm parameters were compared between the heavy smokers and non-smokers, as shown in Table 1. The total sperm count, total motility, progressive motility, normal form, vitality, and sperm membrane integrity were significantly higher in the non-smokers group in comparison to the heavy smokers group: 52.38 ± 34.52 vs. 64.42 ± 39.18 Mill/mL, *p* ≤ 0.01; 44.61 ± 22.47 vs. 53.67 ± 20.51%, *p* ≤ 0.003; 33.17 ± 21.87 vs. 40.96 ± 20.78%, *p* ≤ 0.01; 17.22 ± 8.26 vs. 22.98 ± 12.62%, *p* ≤ 0.05; 59.95 ± 13.08 vs. 64.87 ± 15.18%, *p* ≤ 0.001; and 72.19 ± 10.03 vs. 78.73 ± 11.21%, *p* ≤ 0.02, respectively. There was an exception for immotile sperm, which was significantly higher in the heavy smokers group (51.13 ± 24.23 vs. 42.21 ± 18.24%; *p* ≤ 0.002).

### 3.2. Quantification of mRNA

The quantification Real-time PCR was used to quantify the expression levels of the selected genes (*PGAM5*, *TYRO3*, and *PTPRN2*). The CT represents the threshold cycle and provides information about the cycle of the fluorescent signal that increased exponentially to cross the threshold.

The relative amounts of the *PGAM5*, *TYRO3*, and *PTPRN2* mRNA delta Ct (ΔCT) were differentially expressed among the compared groups (Table 2).

This difference between the group of non-smokers and group of heavy smokers was significant for *PGAM5* (*p* ≤ 0.03) and *PTPRN2* (*p* ≤ 0.01), but not significant for *TYRO3* (*p* ≤ 0.3).

For the *PGAM5* gene, a down-regulation with a 0.57-fold change was determined. Similarly, for the *PTPRN2* gene, there was a down-regulation with a fold change equal to 0.66. In contrast, an upregulation with a 1.21-fold change for the *TYRO3* gene was determined (Table 2).

### 3.3. Correlation between PGAM5, TYRO3, and PTPRN2 mRNA Expression and Sperm Parameters

The association between the expression levels of PGAM5, TYRO3, and PTPRN2 and the sperm parameters was estimated.

The data included in the present study were not normally distributed (non-parametric), therefore, the spearman correlation coefficient (Spearman Roh) was used. Table 3 demonstrates that the PGAM5 mRNA expression level was correlated negatively with motility (r = −0.336, *p* ≤ 0.005) and progressive motility (r = −0.274, *p* ≤ 0.01), and positively with vitality (r = 0.253, *p* ≤ 0.02).

The TYRO3 mRNA expression level showed a significant negative correlation with sperm count (r = −0.331, *p* ≤ 0.009) and vitality (r = −0.230, *p* ≤ 0.04), and a negative correlation with motility (r = 0.339, *p* ≤ 0.008), progressive motility (r = 0.308, *p* ≤ 0.01), normal form (r = 0.259, *p* ≤ 0.04), and sperm membrane integrity (r = 0.269, *p* ≤ 0.01) (Table 3).

Furthermore, the PTPRN2 mRNA expression level was significantly negatively correlated with total sperm count (r = −0.273, *p* ≤ 0.01), motility (r = −0.276, *p* ≤ 0.01), progressive motility (r = −0.293, *p* ≤ 0.009), normal form (r = −0.228, *p* ≤ 0.04), and vitality (r = −0.344, *p* ≤ 0.02) (Table 3).

## 4. Discussion

Infertility or subfertility is the result of many pathological factors. In general, 50% of infertility cases are attributed to idiopathic subfertility. About 15 percent of these cases are due to genetic defects, with the remainder being due to environmental and lifestyle factors such as diet, alcohol consumption, physical activity, and smoking.

Smoking is widely recognized as a major risk factor for various health conditions, including male infertility. The detrimental effects of smoking on fertility are multifaceted and involve several mechanisms [42,43]. The genotoxic components of tobacco cause DNA adduct crosslinks, single- or double-strand breaks, chromosomal abnormalities and aneuploidy, and other genetic changes in the spermatozoa [30].

One significant pathway through which smoking affects male fertility is the induction of DNA adducts, particularly benzopyrene (BaP) DNA adducts. BaP, a polycyclic aromatic hydrocarbon (PAH) found in tobacco smoke, forms covalent adducts with DNA, interfering with DNA replication and repair processes and leading to DNA damage and mutations. These adducts can also disrupt the RNA metabolism by altering the transcription and processing of RNA molecules. Such disruptions in the DNA and RNA metabolism have been associated with impaired sperm production, DNA damage, and reduced sperm quality, contributing to male infertility [44,45].

Additionally, smoking-induced oxidative stress plays a crucial role in male infertility. Tobacco smoke contains harmful chemicals that generate reactive oxygen species (ROS), leading to oxidative DNA damage in sperm cells. This oxidative damage includes single-strand and double-strand breaks, as well as modifications such as 8-hydroxy-2′-deoxyguanosine (8-OHdG) [17,46,47]. The presence of oxidative stress and DNA damage can have profound effects on the sperm DNA methylation, an essential epigenetic mechanism involved in gene regulation. Smoking-related oxidative stress disrupts the DNA methylation patterns in the sperm, resulting in aberrant gene expression and impaired sperm function. This impaired DNA methylation has been implicated in decreased sperm quality and fertility [4,39,48].

In comparison to the control group, we identified a significant decrease in the standard semen parameters (count, total motility, progressive motility, normal form, vitality, and membrane integrity) in the heavy smokers group (*p* ≤ 0.05). These results are consistent with other studies showing the negative effects of smoking on sperm quality and its DNA structure [43,46,48]. A meta-analysis showed that that smoking negatively affects the sperm parameters [20]. Conversely, other researchers have found that tobacco has no impact on the standard semen parameters [49,50,51]. Thus, more research is needed to understand the molecular mechanism of how tobacco smoking influences male fecundity.

This is the first study to focus on a relative quantification of the *PGAM5*, *TYRO3,* and *PTPRN2* genes’ expression and investigate the influence of tobacco smoke on the transcript levels of these genes. The relative amounts of the *PGAM5*, *TYRO3*, and *PTPRN2* mRNA delta Ct (ΔCT) were differentially expressed among the compared groups (Table 2). This difference between the group of non-smokers and group of heavy smokers was significant for *PGAM5* (*p* ≤ 0.03) and *PTPRN2* (*p* ≤ 0.01), but not significant for *TYRO3* (*p* ≤ 0.3).

The *PGAM5* gene was down-regulated in the spermatozoa of the heavy smokers compared to that of the nonsmokers, with a 0.57-fold change. Similarly, for the *PTPRN2* gene, there was a down-regulation with a fold change equal to 0.66. In contrast, an upregulation with a 1.21-fold change for the *TYRO3* gene was determined (Table 2).

These results are in accordance with a study that demonstrated a downregulation in the transcription levels of the *H2BFWT*, *TNP1*, *TNP2*, *PRM1*, and *PRM2* genes in the spermatozoa of heavy smokers compared to that of nonsmokers [48]. Other studies have demonstrated a significant decline in the transcription levels of *PRM1* and *PRM2* in smokers’ spermatozoa compared to that of non-smokers [52], and that reactive oxygen species (ROS) resulting from smoking might be responsible for the decrease in the transcription levels of genes such as IκBα [53]. Another study showed a downregulation in the transcription levels of the *MAPK8IP3*, *GAA*, *ANXA2*, *PRRC2A*, and *PDE11A* genes in heavy smokers compared to non-smokers and an upregulation in the *MAPK8Ip3*, *ANXA2*, *PRRC2A*, and *PDE11A* gene transcription levels in heavy smokers compared to non-smokers [54].

Regarding the correlation between the expression levels of the studied genes and the sperm parameters in the heavy smokers group (Table 3), we found that the *PGAM5* mRNA expression level was correlated negatively with the total motility and progressive motility (*p* ≤ 0.01) and positively with vitality (*p* ≤ 0.02). The *TYRO3* mRNA expression was correlated negatively with the sperm count and vitality (*p* ≤ 0.01), but highly positively with the total motility, progressive motility, sperm membrane integrity (*p* ≤ 0.01), and normal form (*p* ≤ 0.04). In addition, the *PTPRN2* mRNA expression level showed a negative correlation with the total sperm count, total motility, progressive motility (*p* ≤ 0.01), normal form (*p* ≤ 0.04), and vitality (*p* ≤ 0.02) (Table 3).

These results are similar to other studies, which have shown that mutation or downregulation of the *PGMA5* gene can lead to mitochondrial dysfunction, depletion, or a loss of mtDNA [55,56]. Furthermore, these results are consistent with other studies that have reported that *PTPRN2* is located near the hypermethylated regions of sperm DNA from patients who failed to conceive [57]. Another study showed that the *PTPRN2* gene showed highly differential methylation differences between severe oligospermia and obstructive azoospermia [58]. Moreover, a case–control study published in 2019 identified 38 candidate genes, including *PTPRN2*, as being important for spermatogenesis and fertility, which were differentially methylated and/or exhibited a high testes expression between infertility cases and controls [59]. This study also confirmed research suggesting that variations in the *TYRO3* gene may be associated with decreased sperm motility and fertility [36,37,38].

Smoking causes the formation of free radicals, which cause oxidative stress in the body. Oxidative stress can damage the DNA in sperm and lead to a reduced fertility [60]. *PGAM5* has been shown to play a role in protecting cells from oxidative stress [56], so a downregulation of this gene may make sperm more susceptible to damage from smoking-related oxidative stress. In addition, smoking has been shown to impair the mitochondrial function in various tissues, including the sperm [61,62]. *PGAM5* plays a role in regulating mitochondrial function, so a downregulation of this gene may lead to further mitochondrial dysfunction in response to smoking.

Smoking causes inflammation in the body, and chronic inflammation has been linked to decreased fertility. PTPRN2 has been shown to play a role in regulating inflammation. Therefore, a downregulation of this gene may make sperm more vulnerable to the inflammatory damage caused by smoking. Moreover, changes in the transcript levels of these genes confirmed our previous study [39]. Increased DNA methylation levels in the *PGAM5* and *PTPRN2* genes in heavy smokers lead to a repression of the expression of these genes, resulting in the downregulation of the *PGAM5* and *PTPRN2* genes. Conversely, a significant decrease in the DNA methylation (demethylation) levels in *TYRO3* in cases compared to controls could lead to upregulation.

This study confirms that cigarette smoke plays a crucial role in altering the human gene transcription levels in sperm, as it affects chromatin remodeling [63], global DNA methylation status [53], and DNA methylation at different CpG sites [8].

## 5. Conclusions

Overall, this study showed that cigarette smoking may affect the PGAM5, PTPRN2, and TYRO3 genes by altering their DNA methylation patterns. This could alter the sperm quality and consequently male fertility. However, more research is needed, using a larger sample size to fully understand the mechanisms involved and the potential effects of smoking on these genes associated with male fertility. Quitting smoking can improve overall health and fertility and may also help to improve the gene expression and mitochondrial function in sperm.

## Figures and Tables

**Table 1:** Descriptive characteristics of the study population (*n* = 118): comparison between heavy smokers (*n* = 63) and non-smokers (*n* = 55).

Variables	Heavy Smokers (Mean ± SD)	Non-Smokers (Mean ± SD)	*p*-Value
Age	37.42 ± 5.24	36.33 ± 6.18	≤0.4 *
Count (mill/mL)	52.38 ± 34.52	64.42 ± 39.18	≤0.01 *
Total motility (%)	44.61 ± 22.47	53.67 ± 20.51	≤0.003 *
Progressive motility (%)	33.17 ± 21.87	40.96 ± 20.78	≤0.01 *
Immotile (%)	51.13 ± 24.23	42.21 ± 18.24	≤0.002 *
Normal form (%)	17.22 ± 8.26	22.98 ± 12.62	≤0.05 *
Vitality (%)	59.95 ± 13.08	64.87 ± 15.18	≤0.001 *
Membrane integrity (HOS) (%)	72.19 ± 10.03	78.73 ± 11.21	≤0.02 *

SD, stander deviation. * Mann–Whitney test. *p* > 0.05: not significant. *p* ≤ 0.05: significant. *p* ≤ 0.01 highly significant.

**Table 2:** Expression levels of *PGAM5*, *TYRO3*, and *PTPRN2* genes from spermatozoa in heavy smokers compared to non-smokers controls groups (*n* = 118).

Target	ΔCT Heavy Smokers	ΔCT Non-Smokers	ΔΔCT	Fold Change 2 − ΔΔCT	Regulation	*p*-Value
*PGAM5*	3.7 ± 1.6	2.9 ± 1.1	0.804	0.573	Down	≤0.03 *
*TYRO3*	2.3 ± 1.1	2.4 ± 1.0	−0.275	1.209	Up	≤0.3 *
*PTPRN2*	4.5 ± 1.0	3.9 ± 0.9	0.587	0.666	Down	≤0.01 *

* Mann–Whitney test. *p* > 0.05: not significant. *p* ≤ 0.05: significant. *p* ≤ 0.01 highly significant.

**Table 3:** Correlation coefficient between the gene expression level and semen parameters for heavy smokers case group (*n* = 63).

		Total Sperm Count (Mill/mL)	Motility (%)	Progressive (%)	Immotile (%)	Normal Form (%)	Vitality (%)	Membrane Integrity (HOS) (%)
PGAM5	R	0.078	−0.336 **	−0.274 *	−0.001	−0.017	0.253 *	0.020
	P	0.668	0.005	0.015	0.994	0.925	0.026	0.913
TYRO3	R	−0.331 **	0.239 **	0.308 *	−0.218	0.259 *	−0.230 *	0.269 *
	P	0.009	0.008	0.016	0.057	0.045	0.044	0.018
PTPRN5	R	−0.273 *	−0.276 *	−0.293 **	0.268 *	−0.228 *	−0.344 *	−0.194
	P	0.016	0.015	0.009	0.018	0.046	0.021	0.202

Spearman’s test, r: Correlation coefficient. * *p* ≤ 0.05: significant. ** *p* < 0.01 highly significant.

## Data Availability

The data presented in this study are available on request from the corresponding author. The data are not publicly available due to ethical restrictions.
